# Andes Hantavirus-Infection of a 3D Human Lung Tissue Model Reveals a Late Peak in Progeny Virus Production Followed by Increased Levels of Proinflammatory Cytokines and VEGF-A

**DOI:** 10.1371/journal.pone.0149354

**Published:** 2016-02-23

**Authors:** Karin B. Sundström, Anh Thu Nguyen Hoang, Shawon Gupta, Clas Ahlm, Mattias Svensson, Jonas Klingström

**Affiliations:** 1 Department of Microbiology, Tumor and Cell Biology, Karolinska Insitutet, Stockholm, Sweden; 2 Center for Infectious Medicine, Department of Medicine, Karolinska University Hospital Huddinge, Karolinska Insitutet, Stockholm, Sweden; 3 Department of Clinical Microbiology, Division of Infectious Diseases, Umeå University, Umeå, Sweden; Division of Clinical Research, UNITED STATES

## Abstract

Andes virus (ANDV) causes hantavirus pulmonary syndrome (HPS), a severe acute disease with a 40% case fatality rate. Humans are infected via inhalation, and the lungs are severely affected during HPS, but little is known regarding the effects of ANDV-infection of the lung. Using a 3-dimensional air-exposed organotypic human lung tissue model, we analyzed progeny virus production and cytokine-responses after ANDV-infection. After a 7–10 day period of low progeny virus production, a sudden peak in progeny virus levels was observed during approximately one week. This peak in ANDV-production coincided in time with activation of innate immune responses, as shown by induction of type I and III interferons and ISG56. After the peak in ANDV production a low, but stable, level of ANDV progeny was observed until 39 days after infection. Compared to uninfected models, ANDV caused long-term elevated levels of eotaxin-1, IL-6, IL-8, IP-10, and VEGF-A that peaked 20–25 days after infection, i.e., after the observed peak in progeny virus production. Notably, eotaxin-1 was only detected in supernatants from infected models. In conclusion, these findings suggest that ANDV replication in lung tissue elicits a late proinflammatory immune response with possible long-term effects on the local lung cytokine milieu. The change from an innate to a proinflammatory response might be important for the transition from initial asymptomatic infection to severe clinical disease, HPS.

## Introduction

Hantaviruses are the etiological agents of two severe zoonotic diseases, hantavirus pulmonary syndrome (HPS, also called hantavirus cardio-pulmonary syndrome, HCPS) in the Americas, and hemorrhagic fever with renal syndrome (HFRS) in Eurasia, with case fatality rates of 40 and up to 15%, respectively [1–4]. Hantaviruses are normally transmitted to humans via inhalation of virus-contaminated excreta from the rodent hosts [1–4]. In addition, the HPS-causing Andes virus (ANDV) has a potential for person-to-person transmission [5–8]. The lung is the first target for hantaviruses and pulmonary involvement is a major part of the pathogenesis, especially during HPS [2, 9–10]. However, little is known regarding possible hantavirus-mediated effects on the lung.

Endothelial cells are the main targets of hantaviruses in patients, but also epithelial cells are infected [1–4, 11]. Viral antigen has been detected in the upper respiratory tract epithelial cells in ANDV-infected Syrian hamsters, suggesting the possibility that epithelial cells might facilitate transmission of ANDV to the respiratory tract [12]. However, whether replicating hantaviruses are produced by these cells, or if lung epithelial cells are involved in the observed human-to-human spread of ANDV has not been studied.

As lung tissue contains epithelial cells and fibroblasts that interact with each other, the effects of hantavirus infection of lung tissue might not be possible to reproduce in cell culture experiments using only lung epithelial or only fibroblast cells. However, recent advances in creating 3-dimensional organotypic models have facilitated studies on tissue inflammation and infection under physiological conditions [13–17]. Here, we utilized a previously described 3-dimensional organotypic human lung tissue model that includes a differentiated epithelial cell layer and an underlying fibroblast matrix layer reproducing important features of the human lung [13–14] to study ANDV infection.

Our results revealed a sudden peak in ANDV progeny virus production approximately 10 days after initial infection, followed by long-term changes in cytokine levels. These findings suggest that ANDV affects the local lung tissue cytokine milieu, thereby causing a proinflammatory state that might contribute to the-pathogenesis of HPS.

## Material and Methods

### Cells and virus

The human lung fibroblast cell line MRC-5 (ATCC CCL-171) were grown in Minimum Essential Medium (MEM) supplemented with 10% FBS, 100 U of penicillin/ml and 100 μg of streptomycin/ml. The human bronchial epithelial cell line 16HBE14o- [18], a kind gift from Dr. Dieter Gruenert, Mt. Zion Cancer Center, University of California, San Francisco, CA, were grown in Dulbecco´s Modified Eagle´s Medium (DMEM) supplemented with 10% FBS, 1% HEPES, 1% non-essential amino acids, 100 U of penicillin/ml and 100 μg of streptomycin/ml. All reagents were from Life Technologies.

ANDV was propagated and titrated on Vero E6 cells (ATCC Vero C1008) as previously described [19]. The lower limit of detection in the titration assay [19] is 5 focus forming units (FFU) per ml (FFU/ml).

### Preparation of lung tissue models

Lung tissue models were prepared (S1 Fig) essentially as previously described [13–14]. In detail, to set up a lung tissue model, the inner chamber of a six-well transwell insert (Becton Dickinson) was coated with a solution of bovine type I collagen (1.1 mg/ml; Organogenesis) in DMEM and incubated for 30 min at 37°C in 5% CO_2_. A suspension of MRC-5 cells and collagen (230000 cells per ml diluted in 1.1 mg/ml of collagen DMEM mixture) was added onto the precoated collagen layer and incubated for 2 hours. Following polymerization, 2 ml medium were added to the outer chamber, and the culture was incubated for 24 hours. Medium was then removed from the outer and inner chambers, and 2 ml of fresh medium were added to both the inner and the outer chambers. To allow for the fibroblast collagen matrix layer to be formed, the model was then incubated for 7 additional days. All medium was then removed from the chambers and 1.5 ml medium were added to the outer chamber. Next, 50000 16HBE14o- cells in 50 μl medium were added to the fibroblast collagen matrix and incubated for 2 hours. After incubation, 1.5 ml medium were gently added to the insert. The submerged culture was then incubated for 3 days to allow for the epithelial cells to form a confluent layer on the fibroblast collagen matrix. After this step the tissue model was air-exposed by removing the medium from the insert and reducing the volume to 1.5 ml in the outer chamber. Air-exposed models were further incubated for 7 days before infection, and medium was changed every second day.

### Infection and sampling

Lung tissue models were infected with 500000 FFU of ANDV or left uninfected as a negative control. ANDV was added to the air-exposed apical side of the model. Infection was carried out as follows: ANDV was diluted in Hanks’ Balanced Salt Solution (HBSS) supplemented with 100 U of penicillin/ml, 100 μg of streptomycin/ml, 2% FBS and 2% HEPES. Fifty μl of the virus solution was then carefully added to the apical, air-exposed, side of the models.

Supernatants were collected from the basolateral side of the model over time until termination of the experiments. Some models were harvested at time points as indicated in the text for analyses of RNA and protein levels and for apically released viruses. For sampling of apically released virus, the apical side of models was washed with 50 μL PBS which was then collected.

All collected samples were analyzed in duplicate in the specific assays described below.

### LDH assay

A lactate dehydrogenase (LDH) assay (CytoTox 96 Non-Radioactive Cytotoxicity Assay; Promega) was used to measure cytotoxicity according to the manufacturer’s protocol.

### Analyses of cytokine and nitric oxide levels

Enzyme-linked immunosorbent assays (ELISAs) for quantification of IP-10 (CXCL10), RANTES (CCL5), eotaxin-1 (CCL11), VEGF-A, IL-8 (CXCL8) (all Peprotech), TNF and IL-6 (both Mabtech), were performed according to the manufacturer’s protocols.

The production of nitric oxide (NO) was measured indirectly in cell culture supernatants by determination of the level of nitrite using the Griess assay as previously described [20].

### Quantitative RT-PCR

Total RNA from lung tissue models was isolated using TriPure Isolation Reagent (Roche Applied Science) and treated with Turbo DNA-free (Ambion, Life Technologies) to remove any contaminating DNA. cDNA synthesis was performed using Superscript III Reverse Transcriptase (Life Technologies), random primers (Life Technologies) and RNAseOUT Recombinant Ribonuclease Inhibitor (Life Technologies).

Total RNA was measured with TaqMan gene expression assays for human interferon (IFN)-α, IFN-β, IFN-λ1, IFN-λ2, ISG56, RANTES, VEGF-A, and β-actin (all Life Technologies). The forward primer 5’-CAAGAATTGCAGGAAAACATCACA-3’, reverse primer 5’-AGCTTTTGCCGAGCAGTCA-3’, and probe: 5’-CACACGAACAACAGC-3’ (Life Technologies) were used for quantification of negative stranded ANDV S segment (Gen Bank accession number: AF291702.1). Quantitative real time-PCR was performed using ABI7900 HT Fast sequence detection system (Life Technologies). Data were analyzed with software SDS version 2.3. Data were normalized to β-actin and presented as the change in induction relative to that of uninfected cells.

### Immunoblot analysis

Lung tissue models were treated with 1.5 mg/mL collagenase A (Roche Applied Science) for 60 min at room temperature. Collagenase was then inactivated by 5 mM EDTA in PBS. Extracted cells were washed in PBS and homogenized in lysis buffer (150 mM NaCl, 2 mM EDTA, 1% NP-40, and 50 mM Tris, pH 7.6) supplemented with complete protease inhibitor cocktail minitablets (Roche Applied Science). Immunoblot analyses of samples were performed as previously described [21], using mAb 8F3/F3 for detection of ANDV nucleocapsid protein [22], and anti-calnexin mAb (Cell Signaling technologies) for detection of cellular calnexin.

### Immunofluorescence assay

Cells were extracted as described above, washed, transferred to glass slides, dried at room temperature, and fixed in methanol. Detection of hantavirus antigens was performed as previously described [23]. In brief, cells were stained with the anti-nucleocapsid protein mAb 1C12 followed by FITC-conjugated goat anti-mouse IgG (Sigma-Aldrich). Cell nuclei were counterstained with 4´,6´-diamidino-2-phenylindole (DAPI) (Sigma-Aldrich).

### Statistics

Statistical analyses were performed using one-way ANOVA. P-values <0.05 were considered significant. Data are presented as mean ± SEM.

## Results

### ANDV causes a sudden peak in progeny virus production in lung tissue models

**Fig 1:**
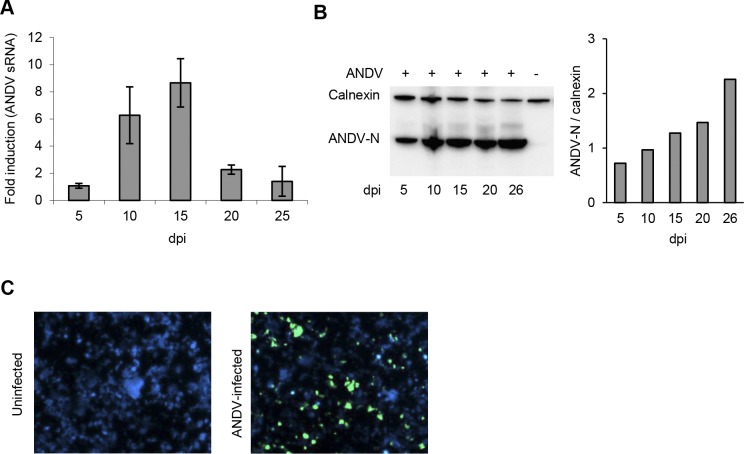
ANDV replicates in the lung tissue model. (A) QPCR analysis of ANDV RNA-levels in cells from lung models over time after infection. Values were normalized to β-actin and presented as fold-change in levels relative to that of day 5 p.i. (B) Representative Western blot showing levels of N-protein over time after infection. (C) Representative immuno-fluorescence images of cells from uninfected and ANDV-infected lung models at day 33 p.i. Data represent mean ± SEM of three independent experiments. In each experiment two infected and two uninfected models were analyzed. dpi; days post infection.

To test if the lung tissue model was susceptible for ANDV-infection, we added ANDV to the air-exposed (apical) side of lung tissue models and then incubated the models for up to 26 days. At 5, 10, 15, 20 and 25 (or 26) days post infection (p.i.), models were terminated, cells extracted, and levels of ANDV S segment RNA and nucleocapsid protein were analyzed. After peaking at day 15 p.i., the ANDV S segment RNA levels decreased (Fig 1A). In contrast, the levels of nucleocapsid protein increased slightly over time up to 26 days p.i. (Fig 1B). Taken together, these findings suggested that the models were successfully infected by ANDV and that ANDV replicated in the models.

New models were then infected and incubated for a total of 33 days. At this time-point cells were extracted from the models and stained for ANDV antigen. The presence of ANDV-infected cells at day 33 p.i. (Fig 1C) shows that ANDV can establish long-term infection in the lung tissue model.

**Fig 2:**
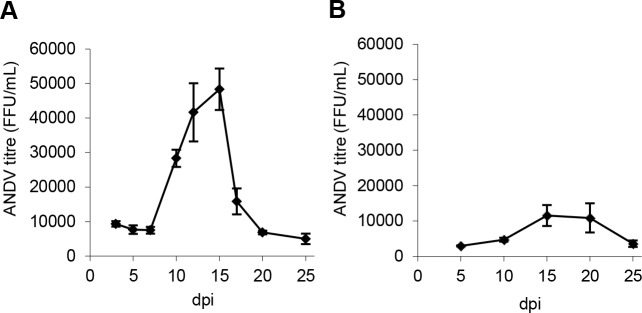
ANDV-infection of lung tissue model results in long-term productive progeny virus production. (A) Levels of progeny viruses detected in basolateral supernatants over time. Data represent mean ± SEM of three independent experiments. In each experiment two infected models were continuously sampled over time until day 25 p.i. (B) Levels of progeny viruses in apical wash. Data represent mean ± SEM of three independent experiments. In each experiment, apical washes from two infected models were sampled 5, 10, 15, 20 or 25 days p.i.. FFU; focus forming units. dpi; days post infection.

In the following experiment, we measured levels of progeny virus in supernatants collected from the basolateral side of infected models. A similar pattern was observed for all analyzed models (Fig 2A). Only low levels of replicating virus were detected at early time-points. A sudden increase of replicating ANDV was observed 7–10 days p.i., followed by high-level progeny virus production for approximately one week (Fig 2A). This peak in ANDV-production (Fig 2A) coincided in time with the peak in ANDV RNA-levels (Fig 1A). Following the peak in ANDV production, low levels of ANDV were observed in supernatants until day 25 p.i. when the experiment was terminated (Fig 2A). To investigate if progeny virus was produced after day 25 p.i., new models were infected and monitored for 39 days. Again, an approximately one-week long period of high-level virus production was observed from day 10 p.i. (S2 Fig). Virus-levels remained low after day 25 p.i., but progeny virus could be detected in the supernatants until termination of the experiment (S2 Fig), showing that viruses are constantly produced in the lung model for at least 39 days p.i..

We then investigated if ANDV was also secreted from the apical side of the model. Indeed, progeny virus was released from the apical side (Fig 2B), with similar time kinetics as observed for the basolaterally released viruses (Fig 2A).

Hantaviruses are not cytopathogenic *per se* [2, 4]; however, possible long-term effects of hantavirus-infection have previously not been studied. To analyze if the decrease in progeny virus production observed from around day 17 p.i. (Fig 2A) was associated with increased cell death in infected models, levels of extracellular LDH in supernatants were analyzed. A slight increase in extracellular LDH was observed in infected compared to uninfected models at day 10 p.i. (S3 Fig). In contrast, from day 15 and onwards, slightly higher LDH-levels were observed in uninfected compared to ANDV-infected models, suggesting that the decrease in progeny virus production observed from around day 17 p.i. was not due to increased cell death caused by the infection (S3 Fig).

### ANDV induces transient type I and type III interferon responses

**Fig 3:**
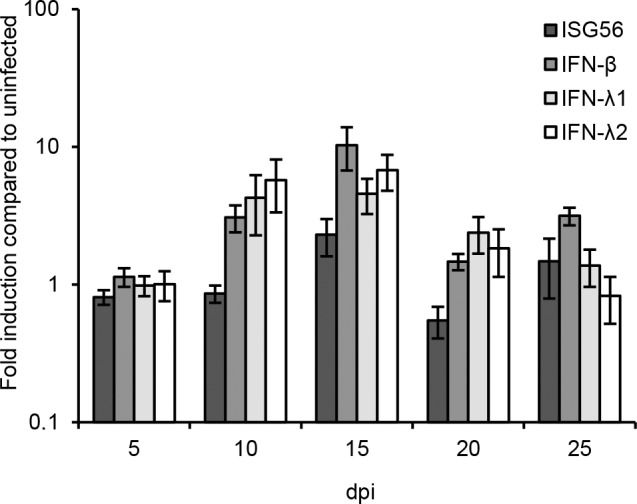
ANDV induces a transient innate immune response in infected lung tissue models. Levels of ISG56, IFN-β, IFN- λ1, and IFN- λ2 and gene expression in lung tissue models. Data were normalized to β-actin and presented as the change in induction relative to that of uninfected models at the same time-point. Data represent mean ± SEM of three independent experiments. In each experiment two infected and two uninfected models were analyzed. dpi; days post infection.

We next analyzed if ANDV induced antiviral responses. Elevated levels of IFN-λ1, IFN-λ2, IFN-β and the IFN-stimulated gene ISG56 mRNAs were detected in ANDV-infected models at days 10 and 15 p.i. (Fig 3), coinciding in time with the observed peak in progeny virus production (Fig 2). Levels of ISG56 and IFN-β mRNA were also slightly increased at day 25. Induction of IFN-α mRNA was not observed at any time-point after ANDV infection.

### ANDV causes elevated levels of the proinflammatory cytokines IP-10, IL-6, and IL-8, and decreased levels of RANTES

**Fig 4:**
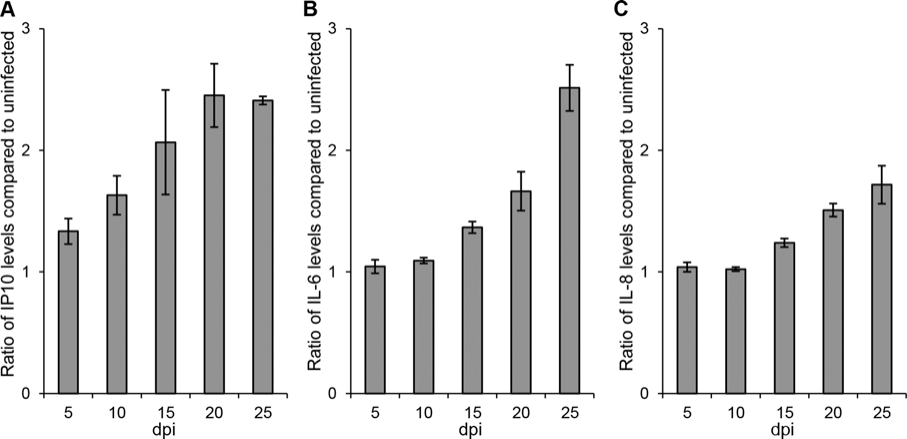
ANDV causes late proinflammatory cytokine responses in lung tissue models. (A) IP-10, (B) IL-6, and (C) IL-8. levels in supernatants over time. Data are presented as relative values of IP-10, IL-6 and IL-8 in supernatants from infected compared to uninfected models. Data represent mean ± SEM of three independent experiments. In each experiment two infected and two uninfected models were analyzed. dpi; days post infection.

Proinflammatory cytokine responses have been suggested to play important roles in HPS pathogenesis [24–25]. We observed significantly (One-way ANOVA; p<0.05) higher amounts of the chemotactic cytokine IP-10 in supernatants from infected models from day 10 p.i. until the end of the experiment (Fig 4A, S4 Fig). Significantly higher levels of the proinflammatory cytokines IL-6 and IL-8 were also observed after ANDV-infection, at day 25 p.i. for IL-6 (Fig 4B, S4 Fig) and at days 20–25 p.i. (Fig 4C, S4 Fig) for IL-8. TNF and nitric oxide (NO) were not detected in supernatants from infected or uninfected models at any time-point.

**Fig 5:**
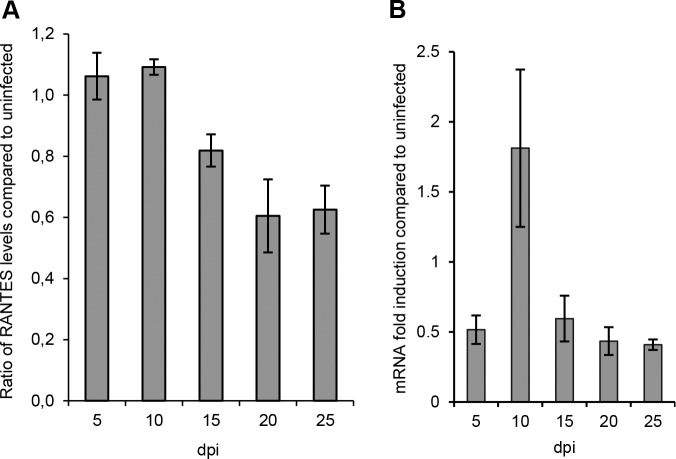
ANDV impacts RANTES levels in lung tissue models. (A) Levels of RANTES in supernatant. Data are presented as relative values of infected compared to uninfected models. (B) Levels of RANTES (CCL5) gene expression in models. Data were normalized to β-actin and presented as the change in induction relative to that of uninfected models. Data represent mean ± SEM of three independent experiments. In each experiment two infected and two uninfected models were analyzed. dpi; days post infection.

In contrast to the elevated levels of IP-10, IL-6 and IL-8, we observed significantly lower levels of the chemotactic cytokine RANTES in supernatants of infected models from day 20 p.i. (Fig 5A, S4 Fig). To analyze if RANTES mRNA-levels were affected by ANDV-infection we analyzed levels of RANTES mRNA over time after infection. Slightly decreased levels of RANTES mRNA were observed in ANDV-infected models at 15–25 days p.i. (Fig 5B), indicating that ANDV-infection can down-regulate RANTES.

### ANDV causes increased levels of eotaxin-1 and VEGF-A

**Fig 6:**
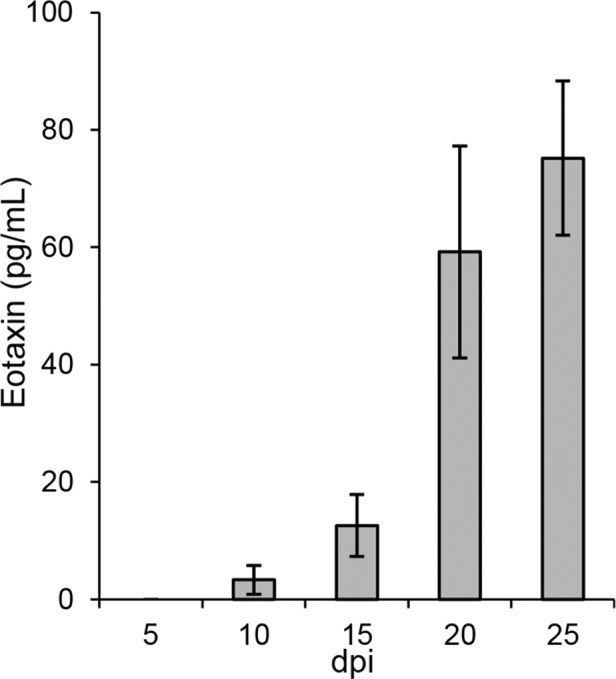
ANDV induces eotaxin-1 in lung tissue models. Levels of Eotaxin-1 in supernatants (pg/ml) from infected models. Eotaxin-1 was not detected in supernatants from uninfected models at any time-point (data not shown). Data represent mean ± SEM of two independent experiments. In each experiment two infected and two uninfected models were analyzed. dpi; days post infection.

Eotaxin-1, a chemotactic protein that mainly acts on eosinophils, has been suggested to play a role in inflammatory lung disorders [26]. This chemokine binds to the same receptor, CCR3, as RANTES [26]. Speculating that also eotaxin-1 could be down-regulated in the infected models, we next analyzed levels of this chemokine in supernatants from uninfected and infected models. Interestingly, while eotaxin-1 was not detected in uninfected models at any time point, it was detected in ANDV-infected models from day 10 p.i. and onwards (Fig 6).

**Fig 7:**
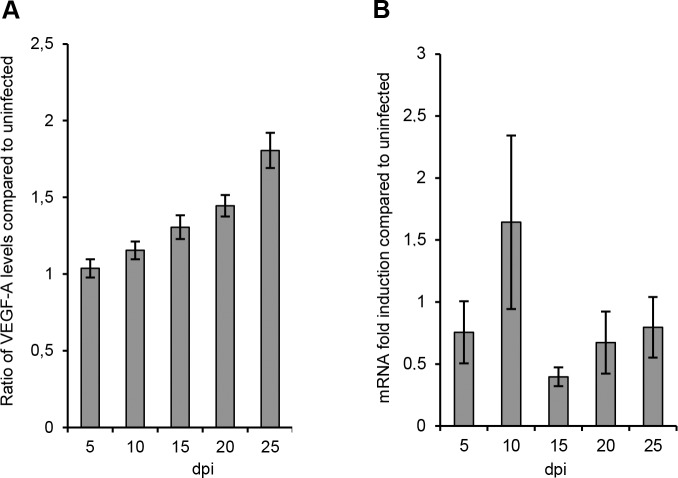
ANDV causes elevated VEGF-A levels in lung tissue models. (A) Levels of VEGF-A in supernatants. Data are presented as relative values of infected compared to uninfected models. (B) Levels of VEGF-A gene expression in models. Data were normalized to β-actin and presented as the change in induction relative to that of uninfected models. Data represent mean ± SEM of three independent experiments. In each experiment two infected and two uninfected models were analyzed. dpi; days post infection.

Elevated VEGF-A levels have been detected in pulmonary edema fluid of HPS patients [27]. We detected significantly increased levels of VEGF-A in supernatants from infected compared to uninfected models late after infection (Fig 7A). However, in contrast to the elevated levels of VEGF-A protein, lower levels of VEGF-A mRNA were observed from day 15 p.i. in ANDV-infected, compared to uninfected, models (Fig 7B), suggesting that VEGF-A is regulated at a post-translational level during ANDV-infection.

## Discussion

Hantaviruses infect humans via the respiratory tract. Since the lungs are severely affected in infected patients, a better understanding of hantavirus mediated effects on the lung tissue may provide new knowledge regarding possible mechanisms behind HPS and HFRS. To study ANDV-infection of human lung tissue we used a recently described 3-dimensional organotypic model of the human lung [13–14] that allows for *in vitro* experiments that cannot be reproduced in monolayers of cells. One important advantage of the human lung model compared to monolayers is the possibility to study infection over a long period of time; *in vitro*, monolayers do not normally support long-term experiments.

ANDV-infection of the air-exposed 3-dimensional organotypic human lung tissue model resulted in a one-week peak in progeny virus production that coincided in time with a transient innate immune response, which in turn was followed by long-term increased eotaxin-1, IL-6, IL-8, IP-10 and VEGF-A, and decreased RANTES, levels. In line with our findings, there are earlier reports indicating a late peak in ANDV progeny virus production. Upon ANDV infection of differentiated primary hamster tracheal epithelial cells, increased levels of progeny virus were observed at day 11 p.i., when the experiment was terminated [28]. In deer mice experimentally infected with ANDV or with Sin Nombre virus (SNV), another HPS-causing hantavirus, viral RNA-levels in the lungs peaked around days 10–15 p.i. [29–30]. Further, in a rhesus macaque-model of HPS, SNV RNA was first detected in whole-blood and serum 12–16 days p.i., preceding respiratory distress and death with 4–10 days [31].

We observed that the peak in progeny virus production coincided in time with the induction of innate immune responses, showing that ANDV-infection causes transient IFN-responses. This IFN-response seems to be able to reduce, although not completely block, ANDV-replication as suggested by the decrease in ANDV S RNA and progeny virus titers observed from 15–20 days p.i. HPS patients show increased proinflammatory cytokine responses, which have been suggested to play a part in the pathogenesis [24–25, 32–33]. Here, we observed clear effects on cytokine levels after the peak in progeny virus production. Interestingly, this suggests a transition from an initial antiviral state into a later proinflammatory response during the ANDV infection. The incubation period of HPS and HFRS range from one to six weeks, with a reported mean incubation period of 18 days for HPS caused by ANDV [1–3, 34]. This long asymptomatic period suggest that initial viral replication is not activating pathogenesis. It is currently not known what marks the transition from asymptomatic hantavirus-infection to HPS. Our data suggest that ANDV infection might cause a switch in the immune response, from an antiviral to an inflammatory response, which might occur when HPS develops. However, it remains to be studied how this local proinflammatory milieu might play a role in HPS pathogenesis by itself, or in combination with other hantavirus-mediated mechanisms, e.g. via recruitment and activation of immune cells.

The finding that ANDV infection has long-term effects on VEGF-A and eotaxin-1 responses is interesting. Elevated VEGF-levels have been detected in pulmonary edema fluid in HPS-patients [26], in serum of HPS and HFRS patients [33, 35–36], and very early after ANDV infection of human primary lung endothelial cells *in vitro* [37]. In contrast to increased VEGF-A levels we observed decreased levels of VEGF-A mRNA. While these findings seemingly contradict each other, it should be noted that VEGF-A translation is regulated on a post-transcriptional level [38–39], indicating that ANDV infection could cause increased VEGF-A translation. Eotaxin-1 binds selectively to the receptor CCR3, which is expressed on eosinophils, basophils and T-helper (Th) cells of the Th2 subtype [26]. Eotaxin-1 is known to activate eosinophils, and eosinophils and their secretory mediators have been suggested to be involved in promoting antiviral host defenses [40]. It remains to be studied if eotaxin-1, and the subsequent activation of eosinophils, have beneficial and/or pathogenic effects during HPS.

## Conclusion

In conclusion, we report that ANDV can cause a late peak in progeny virus production and that ANDV infection results in a complex long-lasting cytokine response in an air-exposed 3-dimensional organotypic human lung tissue model. The finding of a late peak in progeny virus production suggests that ANDV replication may be regulated in a more complex manner than previously known. Further, these findings suggest that ANDV has surprisingly long-term effects on cytokine responses. Interestingly, the observed shift from an antiviral to a proinflammatory milieu late after initial infection might be important for the transition from the initial asymptomatic phase to severe clinical HPS.

## Supporting Information

S1 Fig Schematic representation of preparation of the lung tissue model. Models were prepared as previously described [13–14]. For details, see material and methods.
(PPTX)

S2 Fig ANDV does not cause increased cell death late after infection. Levels of LDH in supernatants were determined by an LDH activity assay. ANDV-infected models were compared to uninfected models at the specific time-points. Data represent mean ± SEM of one experiment with two infected and two uninfected models. FFU; focus forming units. dpi; days post infection.
(PPTX)

S3 Fig Productive progeny virus production for more than 5 weeks after ANDV-infection of lung tissue model. Levels of progeny virus detected in basolateral supernatant over time. Data represent mean ± SEM of three independent experiments. In each experiment two infected models were analyzed. FFU; focus forming units. dpi; days post infection.
(PPTX)

S4 Fig Levels of IP-10, IL-6, IL-8 and RANTES in supernatants from infected and uninfected models. Data are presented as pg/ml. Data represent mean ± SEM of three independent experiments. In each experiment two infected and two uninfected models were analyzed. dpi; days post infection.
(PPTX)
