# Supplementary material for: Andes Hantavirus-Infection of a 3D Human Lung Tissue Model Reveals a Late Peak in Progeny Virus Production Followed by Increased Levels of Proinflammatory Cytokines and VEGF-A
Source: PLoS One. 2016 Feb 23;11(2):e0149354. doi: 10.1371/journal.pone.0149354 (PMC4764364; doi:10.1371/journal.pone.0149354)
Supplement: S1 Fig — Models were prepared as previously described [13–14]. For details, see material and methods. (PPTX) [file pone.0149354.s001.pptx]

## Slide 1
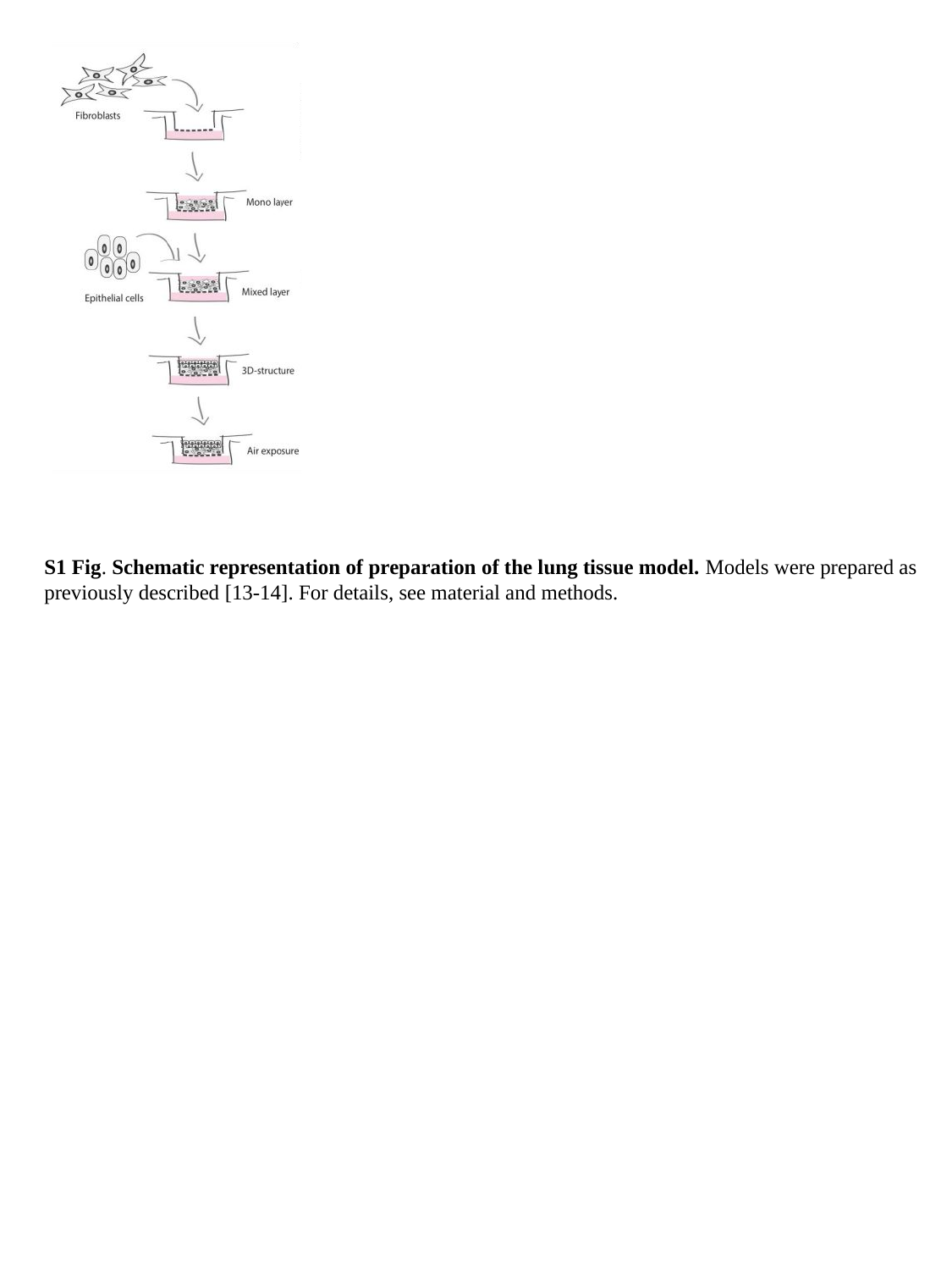

S1 Fig. Schematic representation of preparation of the lung tissue model. Models were prepared as previously described [13-14]. For details, see material and methods.
