# Supplementary material for: Andes Hantavirus-Infection of a 3D Human Lung Tissue Model Reveals a Late Peak in Progeny Virus Production Followed by Increased Levels of Proinflammatory Cytokines and VEGF-A
Source: PLoS One. 2016 Feb 23;11(2):e0149354. doi: 10.1371/journal.pone.0149354 (PMC4764364; doi:10.1371/journal.pone.0149354)
Supplement: S2 Fig — Levels of LDH in supernatants were determined by an LDH activity assay. ANDV-infected models were compared to uninfected models at the specific time-points. Data represent mean ± SEM of one experiment with two infected and two uninfected models. FFU; focus forming units. dpi; days post infection. (PPTX) [file pone.0149354.s002.pptx]

## Slide 1
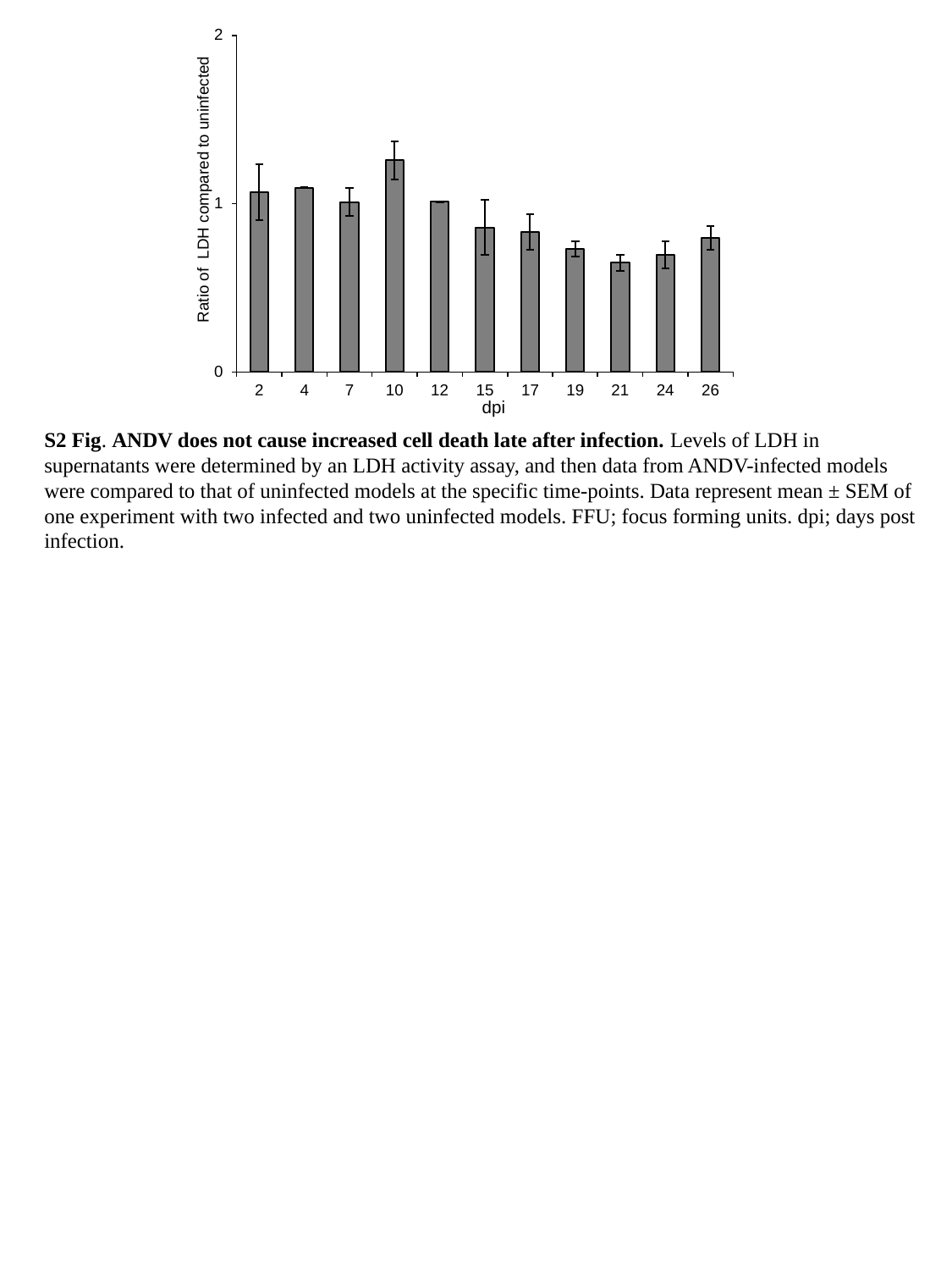

S2 Fig. ANDV does not cause increased cell death late after infection. Levels of LDH in supernatants were determined by an LDH activity assay, and then data from ANDV-infected models were compared to that of uninfected models at the specific time-points. Data represent mean ± SEM of one experiment with two infected and two uninfected models. FFU; focus forming units. dpi; days post infection.
