# Supplementary material for: Andes Hantavirus-Infection of a 3D Human Lung Tissue Model Reveals a Late Peak in Progeny Virus Production Followed by Increased Levels of Proinflammatory Cytokines and VEGF-A
Source: PLoS One. 2016 Feb 23;11(2):e0149354. doi: 10.1371/journal.pone.0149354 (PMC4764364; doi:10.1371/journal.pone.0149354)
Supplement: S3 Fig — Levels of progeny virus detected in basolateral supernatant over time. Data represent mean ± SEM of three independent experiments. In each experiment two infected models were analyzed. FFU; focus forming units. dpi; days post infection. (PPTX) [file pone.0149354.s003.pptx]

## Slide 1
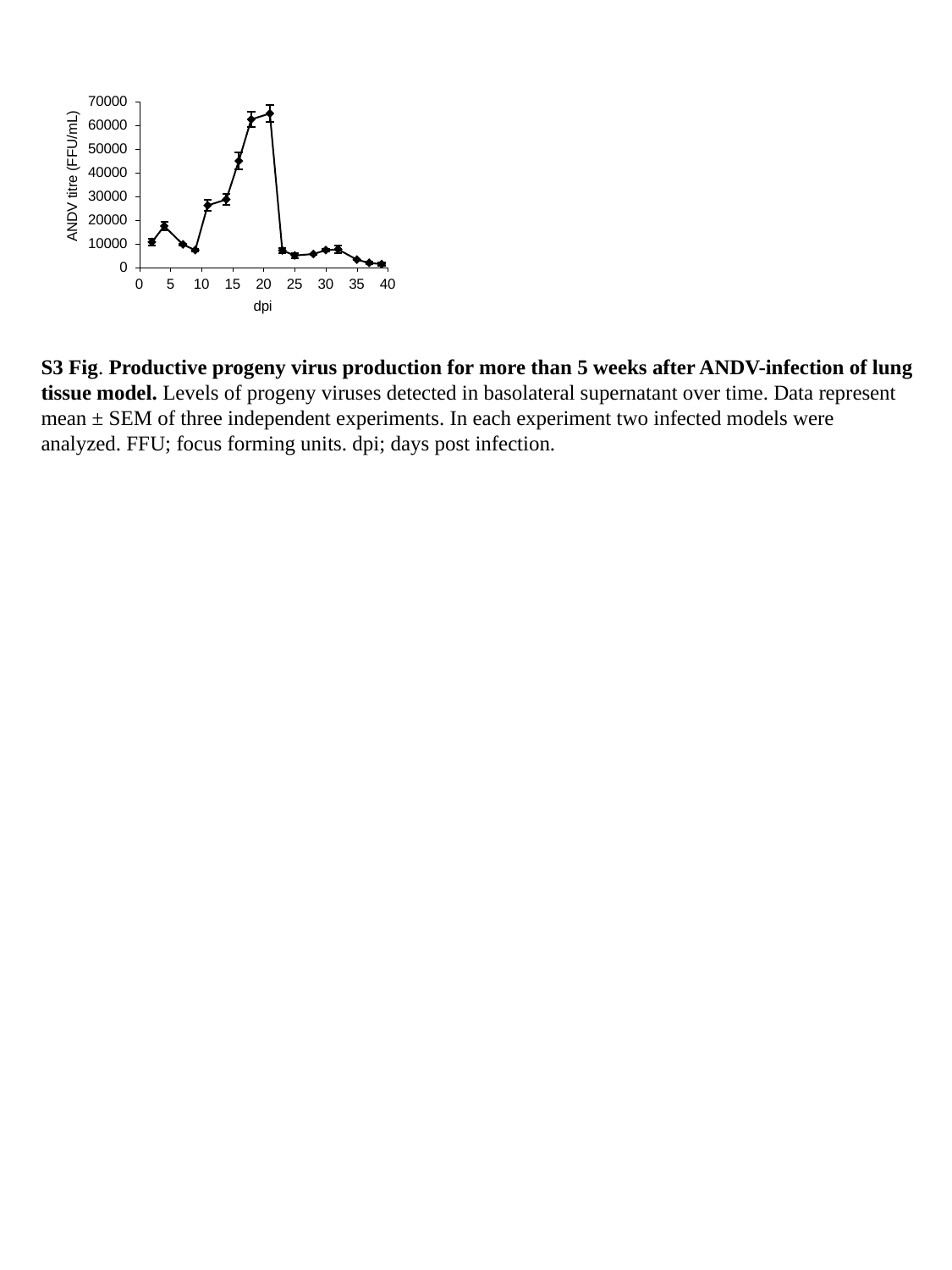

S3 Fig. Productive progeny virus production for more than 5 weeks after ANDV-infection of lung tissue model. Levels of progeny viruses detected in basolateral supernatant over time. Data represent mean ± SEM of three independent experiments. In each experiment two infected models were analyzed. FFU; focus forming units. dpi; days post infection.
