# Supplementary material for: Andes Hantavirus-Infection of a 3D Human Lung Tissue Model Reveals a Late Peak in Progeny Virus Production Followed by Increased Levels of Proinflammatory Cytokines and VEGF-A
Source: PLoS One. 2016 Feb 23;11(2):e0149354. doi: 10.1371/journal.pone.0149354 (PMC4764364; doi:10.1371/journal.pone.0149354)
Supplement: S4 Fig — Data are presented as pg/ml. Data represent mean ± SEM of three independent experiments. In each experiment two infected and two uninfected models were analyzed. dpi; days post infection. (PPTX) [file pone.0149354.s004.pptx]

## Slide 1
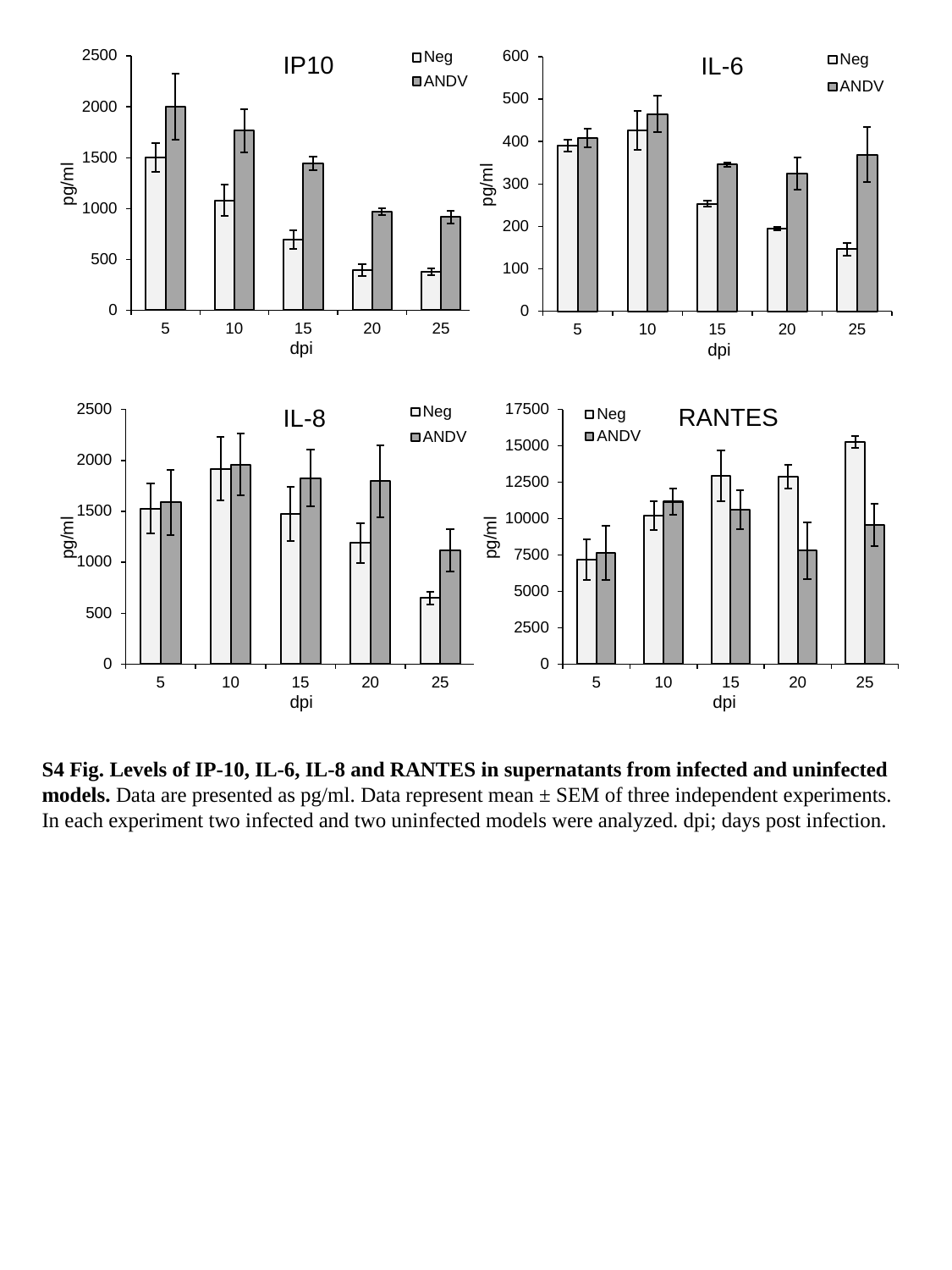

S4 Fig. Levels of IP-10, IL-6, IL-8 and RANTES in supernatants from infected and uninfected models. Data are presented as pg/ml. Data represent mean ± SEM of three independent experiments. In each experiment two infected and two uninfected models were analyzed. dpi; days post infection.
